# Four frames for systemic change in STEM departments

**DOI:** 10.1186/s40594-018-0103-x

**Published:** 2018-02-09

**Authors:** Daniel L. Reinholz, Naneh Apkarian

**Affiliations:** Department of Mathematics and Statistics, San Diego State University, 5500 Campanile Drive, San Diego, CA 92182-7720 USA

**Keywords:** Institutional change, Systemic change, Organizational learning, Culture

## Abstract

**Background:**

This paper adapts the four-frame model of organizational change to the context of higher education. We offer the model as a tool for researchers and change agents who wish to study and enact systemic change within STEM departments. We provide the four frames in contrast to overly simplistic models of change that have been shown to be unlikely to result in sustainable improvements. As we outline the four frames, we discuss both how the frames provide insight into potential products for change and how they influence the process of change. We provide an extended example of how the four frames can be used to analyze an existing change effort and implications of this approach for future work.

**Conclusions:**

This paper adapts a model for promoting and understanding change efforts in STEM departments. This is a model that can be used by nearly any researcher or administrator to help increase the impact of their work.

## Background

Decades of research have resulted in impressive advances in understanding how students learn. There is now strong evidence in favor of active learning, including group work, peer instruction, and “live” response systems (Freeman et al. 2014). While the adoption of active learning techniques has increased over time (cf. Egan et al. 2014), there is still a push to further increase the use of active learning in STEM classrooms (President’s Council of Advisors on Science and Technology 2012). In addition, introductory courses such as calculus remain a major roadblock for STEM students (Bressoud et al. 2013; President’s Council of Advisors on Science and Technology 2012). In other words, while ways to improve teaching and learning are widely known and some progress has been made in their adoption, propagating their widespread use remains a challenge.

To address this challenge, governing bodies across the world have recognized the need to scale and sustain the use of new teaching and learning methods, not just develop them (e.g., Commonwealth of Australia 2015; European Parliament 2015; Niss 2011; President’s Council of Advisors on Science and Technology 2012). In the USA, this is echoed in requests for proposals from major funding agencies such as the National Science Foundation. Moreover, the last decade has seen an increase in STEM-specific institutional change efforts in higher education. These include Vision and Change (AAAS 2011), the STEM Institutional Transformation Action Research Project (SITAR; Corbo et al. 2016), and Student Engagement in Mathematics through an Institutional Network for Active Learning (SEMINAL; Association of Public and Land-Grant Universities 2016). Even though there is a wealth of ongoing change work, the field still needs to adapt models from organizational change to the different context of higher education.

Following the lead of others, we focus on departments as key units of change on a university campus (e.g., AAAS 2011; AACU 2014). Departments introduce and socialize students into disciplines, and they shape how courses are designed and delivered (Lee 2007; Lee et al. 2007). While policies (e.g., teaching loads, committee structures), disciplinary norms, and faculty interactions may differ considerably across contexts, they are relatively consistent within a single department. In other words, departments are relatively coherent units of culture. This contrasts typical work in a faculty learning community, for instance, which typically has a number of faculty members from different departments (Cox 2004a). While the individuals may develop as teachers, it is unlikely that any single department will be significantly impacted by such a diffuse effort. Although there are some instances of FLCs operating entirely within a single department, this is less common, due to the difficulties of facilitating FLCs within a single department (Cox 1995, 1996, 2004b). Nevertheless, in instances where FLCs do work in a single department, they may be able to apply the framework described in this paper to help increase their impact.

Consider some of the goals that a department might have: increasing student success rates, infusing more active learning, developing more coherence across the curriculum, and/or improving diversity in the student body and in faculty positions. How does a department achieve these goals? What questions should it ask? How does it come up with a functional and realistic change strategy? While the educational research literature sheds light on a number of possible “solutions” that a department might seek, there is less guidance on sustainable implementation. However, the organizational change literature has much to offer in guiding a department to achieve its desired ends.

This paper provides such guidance by adapting the four-frame model of organizational change (Bolman and Deal 2008) to the context of higher education. We provide an overview of the frames themselves, define departmental culture in terms of the four frames, and illustrate how the frames speak to the *product* and *process* of change through an extended example. This example is drawn from an existing study that was *not* informed by the four frames but was informed by the change literature generally. Thus, by providing this example, we give an instance of how the four frames can be used as an analytic tool for understanding change. In what follows, we write to support *change agents*: anyone who wishes to study or enact change.

## A brief review of prior change efforts

Change in higher education is a rapidly growing field; a recent meta-analysis reviewed 191 STEM change efforts from 1995 to 2008 alone (Henderson et al. 2011). Yet, most change efforts are guided by overly simplistic, often implicit, theories of change (Borrego and Henderson 2014). Indeed, the authors of this meta-analysis concluded that narrow approaches (e.g., dissemination; top-down mandates), which were nearly 60% of efforts reviewed, were “clearly not effective” (Henderson et al. 2011). A typical folk theory of change is “if you build it, they will come.” At first glance, this is not totally unreasonable. When a new mathematical conjecture is proven, there may be initial debate as to the validity of the proof but upon community validation, the debate ends. Similarly, in the physical sciences, when new techniques are developed, they are quickly adopted by labs across the world. Following this logic, one would similarly expect that active learning techniques and curriculum that support active learning approaches would be adopted in a widespread fashion, but empirical evidence shows otherwise (Apkarian and Kirin 2016; Henderson et al. 2012).

This logic of inference is a naïve interpretation of the theory of diffusion of innovations. That theory describes how early adopters will be the first to engage in new practices, the conditions under which use will continue to spread, and how once a “critical mass” of adopters has been reached, the use of an innovation will be sustained (Rogers 2010). This approach has been used in thousands of studies across various academic disciplines, which have various conceptions of the theory (Greenhalgh et al. 2005). Theoretical issues aside, empirical evidence shows that dissemination or “scale up” approaches have a dubious record in achieving widespread, sustained change in education (Austin 2011; Fairweather 2008; Kezar 2011). For instance, in one national survey on the diffusion of active learning techniques amongst physics faculty, it was found that one third of faculty gave up using active learning after trying just one technique (Henderson et al. 2012). In other words, change in higher education is different from the acceptance of a proof or new laboratory technique. Academic departments are complex social systems, and even widespread terms like “active learning” lack a common meaning across contexts. Furthermore, changing one’s teaching practice to a more student-centered approach is complex and requires not only content knowledge and teaching experience but a shift in the roles of teacher and student and a valuation of “wrong” answers (Speer and Wagner 2009). Enacting and sustaining educational change requires a systemic approach, focused on culture, as we elaborate below.

Even for systemic, department-based efforts, sustainability can be an issue. Consider the Science Education Initiative (SEI), which focused on improving education in science departments (Chasteen et al. 2016). The SEI had three key features—(a) providing expertise and time, (b) working directly with departments, and (c) focusing on individual courses. SEI support was through a competitive grant process, and funded departments received approximately $650,000 over 5 years. This money was used primarily to hire science teaching fellows (STFs), who were postdocs with STEM disciplinary PhDs that received training from SEI leaders. STFs served as educational experts, directly embedded in the departments. The SEI is widely considered successful and has been used as a model for change efforts at number of other institutions. Despite this success, when resources were removed, sustainability became an issue, and course transformations did not necessarily persist (Chasteen et al. 2015). This points to the need for a systemic approach beyond course transformation that seriously considers larger systemic factors and sustainability from the outset. We now outline the four frames, which can be used to inform such an approach.

## The four frames

Culture is hard to define, understand, and measure, but it is a critical component of deep and sustainable change (Schein 2010). To support cultural change, we adapt the four-frame model (Bolman and Deal 2008), which was initially developed for studying businesses. Here, we focus and adapt their model to university departments and provide specific uses for individuals promoting change in such settings. This section describes the four frames (structures, symbols, power, and people) in depth. We begin by defining culture as follows:

Culture is a historical and evolving set of *structures* and *symbols* and the resulting *power* relationships between *people*.

This definition connects the four frames as four interrelated components of culture. We emphasize that departmental culture is not a static object existing in a vacuum. It is embedded within broader society, the discipline, and the university itself. Culture continuously evolves over time and remains tied to the historic development of the department and its past culture. In this sense, cultural change is inevitable (e.g., in response to external stimuli), but it should be the goal of a change agent to modify culture in particular ways, in order to support meaningful change. Furthermore, culture is not monolithic, and departments may have multiple subcultures that change agents should understand and attend to. How different individuals view the culture in a department also depends on their own unique perspectives, so it is difficult to say that there is one coherent culture. Nevertheless, there is some commonality between how individuals in a department interact, and it is important to understand that commonality as well as areas of difference.

The four frames speak to both *what* one may try to change (i.e., the product) and *how* they go about changing it (i.e., the process). In the subsections that follows, we introduce each of the frames, provide a brief description of one insight it provides related to the product of change, and provide one insight related to the process of change.

### Structures

Structures are roles, responsibilities, practices, routines, and incentives that organize how people interact. In an academic context, structures manifest in formal positions (e.g., faculty vs. staff), committee structures, course assignments, research expectations, travel support, class format, common curriculum, and myriad other policies. Structures may manifest at the department level or from other aspects of the institution. For instance, tenure and promotion are critical policies that originate from the college and highest levels of the university itself. While often idiosyncratic, structures within a department are impacted by both the discipline of the department and the institution type. For example, research-intensive institutions tend to have lower teaching assignments than other institution types. Similarly, within a research university, certain disciplines might have lower teaching loads than others or different definitions of research-active faculty. Structures define roles and set expectations for individuals within a department, enabling and constraining their actions. The policies and procedures embodied in a department’s structures are lasting, established aspects of culture that (sometimes implicitly) indicate what is considered important.

Sustainable change requires attending to and changing the structures that define a department. This is best achieved by focusing on broad, positive outcomes to be achieved (Cooperrider et al. 2008; Elrod and Kezar 2015). This contrasts a focus on problems, in which individuals tend to fixate on their preferred solutions, resulting in inflexibility. Such a focus on problems often leads to a disparate set of “fixes” that do not contribute to a coherent whole. In contrast, a focus on outcomes changes the nature of the conversation, allowing group members to see possibilities where before they saw only obstacles. For instance, suppose a faculty group wants to solve the problem of low student engagement in lectures. From a perspective focused on problems, a single group member may fixate on the use of real-time response systems as a solution in a way that derails the progress of the group. If that strategy does not solve the problem (as quick fixes rarely do), it may be abandoned entirely as a failure. In contrast, with a focus on outcomes, the group might instead attend to building strong community and engagement within the student body. This opens the conversation to many other possibilities, including new shared workspaces for students, community events, and awards for faculty who promote student engagement. As part of building a community of engagement, faculty members might consult with students about their concerns and perhaps decide that real-time response systems can not only increase student activity in class but also function as an important part of a feedback loop that improves students’ awareness of their own knowledge and instructors’ awareness of students’ struggles. In contrast to quick fixes, these structural adjustments can support ongoing improvement within a department for years to come.

This frame also emphasizes the importance of incentives and support in the process of change. Tenure and promotion are some of the strongest motivators for faculty, so what is valued in those processes will largely drive faculty behavior. It is also possible for departments to provide modest incentives to promote engagement in a change effort: a summer stipend, travel award, or service credit. These incentives do not carry nearly the weight of tenure, but they are still valuable because they signal departmental support of an effort. If a department is unwilling to commit any resources to an effort, it is unclear if any proposed changes will actually be taken up. Incentives and rewards structures can also be implemented in ways that sustain ongoing change processes. For instance, public acknowledgements or rewards for innovative teaching contribute to a culture of continual improvement; stipends for scholarship of teaching and learning can make it worthwhile and normative for instructors to attend closely to issues in their own classroom; and including discussion of teaching in departmental meetings contributes to normalizing conversations about instruction.

### Symbols

Symbols constitute the cultural artifacts, language, knowledge, myths, values, and vision that department members use to guide their reasoning. While traditional disciplinary representations are also considered in this frame, our use of the term symbols is much broader. Here, *symbols* are the underlying ways of thinking that give meaning to the structures that exist within a department. As an example, consider department meetings. This is a common formal structure that exists across disciplines and institutions. However, their implementation can be quite varied based on how department members value those meetings and understand their meaning. In some departments, all members are expected to attend all meetings and weigh in democratically on all decisions that affect the department. In others, there is not much expectation of attendance and they serve primarily as a way for department leaders to inform their colleagues of decisions that have already been made. The difference in how this structure is taken up relates to the values (symbols) of the department.

Given that symbols dictate how structures are taken up, any structural shift must also be accompanied by a symbolic shift for meaningful change to result. Care is needed in a framing a change effort. For instance, when an effort focuses explicitly on attitudes and beliefs, it may be met with hostility, as this implies that something is wrong with the existing ways of thinking in the department (Schein 2010). Thus, while symbolic shifts are important, they occur most smoothly as part of a larger change effort. Just as instructors use practices to develop classroom norms, departments can develop new structures to help develop new norms (or symbols) in the department. These new structures provide objects for ongoing discussion and reflection. For instance, suppose a department implements a brown bag lunch for talking about teaching. While this could be a place for faculty to complain about students or just trade course materials, it could also serve as a platform for faculty to reflect on their teaching in meaningful ways, which then lead to symbolic shifts (e.g., adopting an asset rather than deficit approach to learning). Intentional decisions about what is presented at such meetings and attention to shaping the conversations can promote the latter.

The change process must also account for the existing cultural artifacts, beliefs, and shared assumptions in a department. In doing so, the effort accounts for where the department presently is, just as modern theories of learning emphasize building on the current knowledge of students (e.g., diSessa 2002; Lave 1996). Given that faculty members are disciplinary experts, there is generally a strong disciplinary identity within the ways of thinking in a group. For instance, most scientists are used to gathering and reasoning with empirical evidence; evolutionary biologists are well-equipped to draw upon complex systems thinking and how that relates to change; and engineers draw upon skills with rapid prototyping and testing. Change agents external to a department must understand these ways of thinking to better understand a change effort.

### People

Departments are composed of people with individual goals, agency, needs, and identities. This frame emphasizes that, while communities have common ground, they are ultimately composed of individuals. For instance, all computer scientists are not the same and may have a variety of different personal and professional commitments, such as to equity or social justice. It is important to attend to the numerous complex and interacting identities of the individuals within a department when considering changes to that department. This frame draws attention to the complex and sometimes divergent perceptions and experiences within a department (i.e., the culture of the department is not the same for all individuals all of the time). Thus, a more complete picture of culture is developed when multiple perspectives, and their idiosyncrasies, are taken into consideration.

In thinking about products of change, the people frame guides change agents to consider solutions that embody a shared vision that attends to the needs, goals, and identities of participants. This allows a group to come together to develop collective goals that attend to, and include, individual goals and concerns. A shared vision for the department can help shape the direction of future change initiatives to align with the needs of individual members as well as build coherence amongst those goals and ideals. It also allows individuals to see areas of commonality between their goals that otherwise may have not been evident.

The people of the department should also be taken into consideration when making process-related decisions about a change initiative. In particular, it is important to consider the agency of individuals within the department. Agency relates to the ability of individuals to influence their circumstances (Bandura 2006). Crucially, agency drives motivation, as it links directly to fulfilling basic human psychological needs (Deci and Ryan 2000). If department members are simply handed a solution and expected to implement it with fidelity, success is unlikely to result. In contrast, when they are given high expectations and high support, with the space to creatively work for improvements, success is much more likely. This means that the successful change agent should work with individuals in the department and support their independence, allowing them to take ownership of innovation efforts. When the people of the department drive the change themselves, they develop personal interest in the success of initiatives, and this is often a strong motivator.

### Power

Within a department, interactions between people are mediated by power, status, positioning, and political coalitions. Power differences may arise in a number of different ways: from formal roles (e.g., department chair vs. associate professor vs. assistant professor), success and status within the field, or other aspects of identity such as race, gender, sexual orientation, or ability status. Power is always at play (Foucault 1977) and cannot be ignored. Existing power structures must be recognized, as they heavily influence people and decision-making. Department-based change may be top-down (administrator-led), bottom-up (faculty- or student-driven), or some combination of the two. Whatever the case, the group that initiates the change effort must involve others early on and in meaningful ways. Otherwise, they may invest a large amount of effort that ultimately does not lead to anything.

It is also an unfortunate fact that the existing hierarchies in academia have marginalized particular groups of people, but change agents can intentionally design solutions that work to disrupt those hierarchies and promote equity. One way that this can be done is by involving traditionally marginalized voices in the development of a shared vision so that more perspectives can be included. Similarly, by creating formal structures that involve other voices (e.g., having students on faculty committees; ensuring that lecturers vote on instructional decisions), it is possible to support a greater distribution of power. Ultimately, these efforts can work to increase equity in a department.

Issues of power affect the processes of change as well. A change effort requires sanction from the appropriate power holders to succeed. Individuals may hold such power due to formal positions in a department or because they are highly respected thought leaders. Because change is time-consuming (Darling-Hammond et al. 2009; Elrod and Kezar 2015; Wilson and Berne 1999), it is key to build in concrete successes, or *early wins* (Kotter 1996) into a change process that signal both to participants and external stakeholders that progress is being made. Without some sign of progress, it is easy to lose support from those who have the power to sanction the change. Consider a group that aims to build greater community for its students. While changing the community and the culture of the department is a many year project, there are many waypoints or markers of change that would provide evidence of improvement. For example, the group could survey students about their experiences, run community events, run faculty professional development for inclusive teaching, or seek vexternal funding. By conducting these activities, the group creates concrete successes toward the larger goal of building community that help maintain external support.

### Summary of frames

We presented the four frames individually to highlight several components of departmental culture so as to help change agents and researchers organize and realize their work. To summarize, *structures* are the roles, routines, and practices of a department; their enactment and meaning are dependent on *symbols*, which are the norms, values, and ways of thinking in a department; changes are ultimately enacted by *people* whose individuality impacts their intentions and perceptions; and the distribution of *power* determines who makes certain decisions and influences interactions. Change efforts must pay attention to all four of these frames and their implications for both products and process; if some of them are ignored, then the sustainability of an effort is dubious (Laursen 2016). These insights are summarized in Table 1. These are not the only possible insights from the framework, but they serve as a productive starting point for individuals new to this literature.

**Table 1:** Product and process of change as informed by the four frames

	Aspects of product	Aspects of process
Structures	A new *thing* that addresses an issue in an *ongoing*, *sustainable fashion*.	Create incentives and support for individuals to engage in the change process and the new “thing.”
Symbols	Attitudes and beliefs that support a proposed change so that it is optimally taken up.	Use language, data, and evidence that align with the department’s present ways of thinking.
People	Solutions that embody a shared vision that attends to the needs, goals, and identity of participants.	Afford individuals the agency to enact *their* change.
Power	Leadership structures that promote equity by attending to the needs of diverse stakeholders.	Use concrete signs of success to develop and maintain the sanction of key stakeholders.

It is also important to think of the coordination of the frames. Indeed, we think of structures and symbols most meaningfully together as a pair. The structures are the visible signs of how a culture works, but the symbols determine how the structures are actually enacted. As such, the two are inextricably linked. Similarly, people and power are related as a pair. The people frame focuses on the importance of individuality, while power draws attention to the way that all of these individuals are linked in a political system. This is not to imply that the frames are not related in other ways, only that we see these pairings as particularly salient. Next, we present an extended example wherein we illustrate the frames in the context of a real department’s change efforts.

## An extended example

### Context

With the four frames in hand, we return to one of the questions posed at the beginning of this paper: how to increase the coherence of a department’s curriculum. We analyze an example from the SITAR project (Reinholz et al. in press) using the four frames to guide our discussion. This project was designed using principles of organizational change and, while the four frames theory was not an explicit aspect of the initial design, we can use this perspective to more clearly understand the effort. The study took place in the context of a STEM department in a research-intensive institution. This particular department (called *Runes* to protect its identity) had previously worked as a part of the SEI and wished to sustain the prior improvements that began to backslide when external support was removed. To support such improvements, a Departmental Action Team (DAT) was formed, which is a particular type of faculty working group that aims to promote systemic change.

Two facilitators from the SITAR project team (at the local institution) worked closely with the Runes DAT, which consisted of five faculty members from the Runes Department: Anne, Bart, Elly, Karen, and Sophia. The four women who were participants were non-tenure track instructors, and there was one man who was a tenured professor. There was extensive data collection in the DAT, including individual interviews, focus groups, artifacts collected, and records from meetings. The Runes DAT met for 16 1-h meetings over the course of the 2014–2015 academic year and continued meeting through the subsequent year. Here, we focus on the first 16 meetings, which related to the development of a new structure in the department; meetings during the second year were primarily to refine what had already been sanctioned by the department. Our analysis of the DAT is limited here, recognizing that this approach has been described in greater depth elsewhere (Reinholz et al. in press).

In Table 2, we present an outline of the product and process of this initiative, as seen through the four-frame model. The new structure that was developed was a set of “curriculum coordinator” positions. The coordinators provide a sustainable mechanism for communication across courses to support a coherent Runes curriculum but do not dictate how courses are taught. We now briefly describe the products and process of change and how they reflect different aspects of the four-frame model.

**Table 2:** Product and process of change in the Runes DAT

	Product	Process
Structures	Develop new curriculum coordinator positions	Course releases for DAT members and coordinators
Symbols	Provide new ways for thinking about deep learning for students in the department	Collected data to make case to the department; acknowledged and addressed known problems
People	Develop a shared vision about curricular coherence and students based around existing concerns	Instructors retain relative pedagogical autonomy; agency for initiatives remained with department members
Power	Coordinators give a voice to instructors and tenured faculty	Ongoing discussions with relevant stakeholders

### The change products

#### Structures

Initially, there was discussion of how the DAT members themselves might work to improve the coherence of the curriculum as a small subcommittee. It soon became clear that the scope of the work was much larger, more stakeholders needed to be involved, and the curriculum was something that need to be revisited on an ongoing basis. Hence, the participants settled on the creation of a new sustainable structure. The overall goal of the coordinators is to facilitate conversations amongst faculty members teaching the required Runes courses to ensure coherence in learning goals and student experiences across them. Each coordinator is responsible for a subset of the major courses and meets with the faculty associated with their set of courses at least once each semester. Collectively, the coordinators collaborate to support coherence across the curriculum and provide instructional support more generally.

#### Symbols

The creation of new coordinator positions was not done in a vacuum. The DAT expended considerable effort developing the messaging and mission of this structure so as to develop attitudes, beliefs, and values (i.e., symbols) to optimize how the coordination system was taken up and sustained. They also developed a framework around student learning with three components: (1) content knowledge, (2) critical thinking, and (3) professional skills. This particular framework was developed to be responsive to the current ways of thinking in the department but also provide greater attention to non-content skills as a way to gradually shift the thinking (i.e., symbols) in the department.

#### People

The ultimate outcome of the DAT represented the collective vision of the DAT members, informed by frequent communications with the department. To reach this end, the initial meetings of the DAT involved members writing down their goals for the department and students, which were sorted into different categories that supported the DAT members to have a common discussion about their goals. This resulted in the decision to focus on curricular coherence, broadly conceived.

#### Power

Finally, the coordinator positions have the potential to shift power structures within the department. The positions are taken up by non-tenure track instructors but have the sanction of the department chair. Moreover, they have the potential to include more voices in how classes are taught because teaching becomes a collective conversation rather than only an individual private act.

### The change process

#### Structures

When the chair of the Runes department was approached with the idea of forming a DAT, from the outset they were willing to offer some support in terms of incentives. In particular, the chair allocated a course release for one of the DAT participants who was supposed to serve as a liaison between the SITAR team and the department. This DAT member took on some additional responsibilities compared to the other faculty members, such as reserving rooms and dealing with other logistics. In addition, when the curriculum coordinator positions were developed, they were sanctioned with three course releases per year (one release per instructor). The inclusion of incentives and rewards for participation in the DAT and coordination system as part of the process is aligned with the structural frame.

#### Symbols

Up until this point, the Runes department had not put much effort into systematically collecting data about students’ course taking patterns and success. In terms of the symbols’ frame, this is indicative of the attitudes of the department about the value of data. Due to this, initial discussions in the DAT were based on anecdotes about students in the major taking courses out of order, since there was no system for enforcing prerequisites. To enact a symbolic shift that gave more importance to rigorous data, DAT members collected and analyzed institutional data related to students’ course taking patterns. As this was data about widely held suspicions in the department, it was aligned with existing ways of thinking and therefore relevant to a broad audience. This attention to the department’s existing values as part of the change process is aligned with the symbolic frame.

#### People

One critical feature of the DAT is that it was composed of department members, in this case five faculty. While the SITAR team worked with them, offering suggestions and guidance, it was ultimately the DAT who chose what course of action to pursue. Thus, agency and ownership of the initiatives were squarely theirs. It was the DAT faculty members who settled on curricular coherence as a common goal worth pursuing, and this personal interest in the outcomes of the initiatives motivated their continued efforts. This internal ownership of the change initiative is aligned with the people frame.

#### Power

The DAT’s analysis of institutional data corroborated their original ideas and provided a concrete source of data to illustrate the problem to the department when asking for support from those with power over structural and financial decision-making. In particular, the facts that students had extremely variable experiences in the major and that it was very hard for courses to build on one another, helped make the case for formal positions to continually improve the coherence of the curriculum. To maintain departmental support for the effort, DAT members had frequent meetings with relevant stakeholders in the department on other committees and also stayed in communication with the department chair. This also allowed the DAT members to gauge external support for the ideas they were thinking to propose, before actually going through the formal process of presenting ideas at a faculty meeting. The attention to stakeholders and those with power in the department is aligned with the power frame.

## Discussion

While seemingly simple, these principles have the potential to impact how an effort proceeds. For example, a focus on creating new structures could help participants get out of quick-fix mode to build positive outcomes. To understand how structures may be built and enacted, a change agent could draw upon the symbolic frame. Similarly, attending to the people involved in an effort and the power relations between them may support the inclusion of relevant stakeholders and their sustained engagement in the process.

We opened the paper with a number of goals a department may have, such as increasing the coherence of its curriculum or the use of active learning. The extended example above showed how the frames can be used to understand such a change effort. The frames could also play a role in the early planning of a change effort, by helping a change agent determine some relevant questions that they should ask (see Table 3). Answering these questions would help a change agent get a better sense of a department and its local culture, which ultimately could be used to inform the nature of the change effort. One could then begin to develop a collective shared vision for the future of the department, identify structures that need to be created or amended, and determine attitudes and beliefs that may impact how things are taken up and by whom.

**Table 3:** Key questions raised by the four frames

Structures	Symbols
• What key structures determine department functioning?	• How do data inform decision-making?
• Do existing structures support new goals?	• What are shared assumptions and values?
• What incentives and supports exist?	• What constitutes evidence?
People	Power
• Who has agency?	• What power hierarchies exist?
• What are their goals?	• Is there support for change?
• What identities do individuals have?	• How is progress communicated and coalitions built?

As it is evident in Table 3, the four frames also speak to general ways of thinking and interacting that can also be implemented in other aspects of a department’s functioning, such as the way that committees are run and how faculty collaborate. Even for smaller change efforts, such as working with a single faculty member, we believe that these principles are still informative. Focusing on structures, affording agency to the faculty member (rather than trying to change them), and mitigating power differentials can all help support improvement.

## Conclusion

Change is notoriously difficult but also urgently needed in higher education. Here, we have adapted the four-frame model as a theoretical tool to support and study sustainable systemic change. This model has implications as a tool for change agents to guide new change efforts and as an analytic lens for researchers to study the change process. While using the four-frame model in no way guarantees the success of an effort, attending to its features increases the likelihood of changes being sustained by ensuring that multiple aspects of culture are being taken into account. We urge the STEM education community to pay serious attention to culture as a focus of change, as extensive research draws attention to the perils of ignoring culture (e.g., Austin 2011; Fairweather 2008; Kezar 2011). This is embodied in the common wisdom of organizational learning, in the phrase, often attributed to Peter Drucker, “culture eats strategy for breakfast.”

The prospect of change in undergraduate STEM education is promising. While there have been global calls for action for years, the advances of the education research community and the adaptation of organizational learning theories to higher education may well increase the likelihood of success. We believe that STEM departments across the world are ready to take new steps to increase interest and success in STEM and present the four-frame model for change to support meaningful, systemic, sustainable improvements.
